# Research on Perceptual Integrity of Distributed Digital Media Based on AWTC-TT Algorithm Optimization

**DOI:** 10.1155/2022/9675529

**Published:** 2022-07-19

**Authors:** Xiaobo Zhang, Rongrong Shen

**Affiliations:** ^1^School of Art, Shandong Jianzhu University, Jinan 250101, China; ^2^School of Law, Shandong Jianzhu University, Jinan 250101, China

## Abstract

Digital media can spread data and information. Its wide use not only enriches people's work and life but also brings high efficiency and convenience. However, due to different devices, different data quality, network stability, and other reasons, data quality is usually difficult to guarantee, so it is necessary to evaluate its perceptual integrity. In this paper, the group intelligence sensing technology in the sensor field and AWTC-TT optimization algorithm integrating blockchain technology are proposed to collect users' sensing data and build a model to discuss the complete performance. The experimental results show that (1) for the AWTC-TT algorithm, the processing time of 200 data tasks is less than 60 s; moreover, after optimization, the performance is efficient, which can effectively reduce the running time of data processing, and the performance loss is only 4%. (2) The perceptual quality of data was evaluated, the gain value of the first 0.4 is above 0.3, and the interference of abnormal data on integrity was explored. (3) The test results of the four integrality indexes of the model are good and the quality is stable. (4) The income value of CS2 is 5.8, and the income of distribution with the highest quality is the highest. Finally, the model constructed in this paper is mature, the experimental data and results are good, and the data perception integrity is discussed. The use of various fusion technologies can provide a reference for more subsequent research and application and lay a solid foundation.

## 1. Introduction

With the rapid changes of the times, many new branches have gradually emerged from the technology with the Internet as the core. With the support of 5G network and computer technology, the idea of connecting and communicating between people and things is no longer a dream [1]. At the same time, a large number of digital media terminal devices enter people's daily life, creating massive data that is difficult to estimate by manpower every day. In this case, a new paradigm of group intelligence perception was born. Through mobile devices and media, people can share perceptual data, which greatly expands the service scope of the Internet of Things. Therefore, the backwardness of some traditional technologies has brought an unbearable burden to the network, and people have to face new problems of data integrity. In addition, the group intelligence awareness service requires high network nodes and speed. Facing challenges, blockchain technology stands out because of its many advantages and outstanding performance in financial transactions. In this paper, the distributed structure of digital media is the main research object, for its perceptual integrity experiments. In this paper, the AWTC algorithm, which is mainly based on blockchain technology, will be optimized, assisted by a consensus algorithm, incentive mechanism, security and trust guarantee, etc. In order to increase the performance of the experiment, the paper also introduces edge computing, the Internet of Things, and blockchain to integrate technologies, so as to reduce some computing pressure and carry out technological reform and integration innovation.

In order to better provide a relevant theoretical basis and experimental data support for our paper, we collected some literature for reference, as follows: Explore the framework and typical application of blockchain technology in energy Internet [2]. Collect data through sensors, study MCS system, and explore many aspects from sensor communication to system management and data storage [3]. Analyze some open source projects of blockchain technology, and deeply analyze the basic principles of blockchain [4]. This paper studies the task allocation problem perceived by mobile people for multitasking participants [5]. Critically evaluate the superiority of blockchain technology, analyze the current situation and look forward to the future of this technology [6]. Learn from the successful experience of blockchain in the application of digital crypto currency and explore the theory of trusted data management [7]. The consistent hash algorithm is used to solve the problems of storage space, repeated verification, and data hierarchical management [8]. Considering 6G and the Internet of Things, the data sharing and storage mechanism of blockchain based on the Gossip protocol is proposed [9]. Objectively expound on the hot spots and trends in the field of integrating blockchain and the Internet of Things at the technical and application levels [10]. A multi-layer blockchain network model for edge computing and an adaptive workload proof algorithm are proposed [11]. The basic network structure of blockchain is realized through various encryption means and consensus algorithms, and the security architecture of edge computing and the Internet of Things is designed [12]. The incentive mechanism based on blockchain technology adopts the distributed architecture of blockchain security and applies group intelligence perception [13]. The transaction information is recorded by a three-tier cognitive radio system based on a blockchain of reputation value auction, and the perceived revenue distribution mechanism is constructed [14]. Based on discrete logarithm and computational Diffie–Hellman proposed a node exit mechanism suitable for a signcryption scheme [15]. This paper studies the influence of blockchain technology on ordinary netizens' perception of public opinion information quality and willingness to accept behavior [16].

## 2. Theoretical Basis

### 2.1. Distributed Digital Media

“Digital media” [17]: it can be interpreted as an information carrier, mainly in the form of binary numbers, which is closely related to computer information technology. However, from a broad perspective, the recording, processing, dissemination, and acquisition functions of digital media have already penetrated into all aspects of the human world. The coverage of intelligent products promotes the advent of the information explosion era, and digital media is widely used by people for its information acquisition and output technology. Its development is no longer only linked with the Internet and IT technology, but with the development of the whole industry and the whole world. Human society can no longer leave digital media technology. Without this technology, the world will lack the energy and driving force to develop the future.

Combine digital technology and media organically. Digital media is usually carried by terminal devices such as TV, mobile phone, computer, and tablet, and output in the form of audio, image, and video. In the output process, it will generate massive data, transmit data according to different goals and needs, and share it with people or things. Not only can human-computer interaction be carried out but also the entertainment interest can be enhanced. Cutting-edge technologies in almost all fields can be integrated with digital media technology, and the digital products created fully reflect the value of commerce and spread culture and art.

Compared with the centralized client/server structure, each network node in the distributed structure has multiple paths, and its database is composed of several related databases on the network. Each network node is not closely related, and they all have the ability to process independently. Therefore, when a node goes wrong, it will not cause the whole system to crash. Moreover, the distributed structure is easy to upgrade and maintain, costs less money, and has multiple servers for local operations. When digital media is combined with distributed architecture, it can effectively divide the number of tasks, improve the efficiency of the whole system, balance the load problem, and run multiple applications at the same time.

### 2.2. Blockchain Technology

BC [18]: it is called “blockchain.” BC can be interpreted as an open distributed digital ledger, which is mainly based on a peer-to-peer network (i.e., P2P network). This ledger can record the information of all transactions, and even every node of the whole network keeps a copy of the ledger. Therefore, only one node is needed, and we can obtain and query the whole transaction data. However, we can't change all the data copies on the whole network by modifying the copy on a certain node. Initially, blockchain technology mainly served the network “mining,” that is, commercial transactions with Bitcoin as the trading currency. With the success of this technology in the financial field, BC has attracted the attention of experts and scholars at home and abroad because of its encryption mechanism, various computing algorithms, consensus mechanism and other technologies. So far, the research on it has become mature. BC technology is not a new single technology, but a new product of the integration of multiple fields and frontier science and technology. It is a continuous and related data system from the perspective of trust, and it is also a decentralized data recording method. Apart from decentralization, most of BC's network data is open and transparent, but users' true identities can be anonymous. Trust is generated between nodes through the hash algorithm. When data is written, in order to ensure the integrity of data, BC can eliminate all modification interventions without authority. Only when you are authorized to control 51% of the nodes can you change the data content or modify the status in BC.

#### 2.2.1. AWTC-TT Algorithm

AWTC [19]: it is called “account-wise transaction chain” in English. It is also better known as “nano block lattice.” Running in the way of winding blockchain, it belongs to a DAG type. It is its existence that enables each account to have a separate blockchain bookkeeping record, which is the core technology of blockchain. Because BC technology is the main medium of economic transactions, it is necessary to ensure fast, safe, effective, and reliable transactions. Although other methods can achieve the above goals, only AWTC can achieve it quickly and stably. Through this algorithm, we can increase the number of network nodes and transactions in the blockchain. While increasing the number steadily, it can also keep the transaction time stable under the maintenance of the algorithm, without being affected by the increasing number of nodes and transactions, thus avoiding other problems. The AWTC algorithm can improve the computational efficiency and provide faster transaction speed for the network and its users. The Locus Chain protocol based on AWTC can upgrade the processing capacity and solve the problem of data scale expansion after introducing dynamic state fragmentation technology. In addition, even in the most stringent network environment, the Locus Chain protocol can support intelligent contracts of blockchain and avoid malicious attacks on the network. After some excellent transformation to improve the performance, we use the AWTC-TT algorithm in this paper.

#### 2.2.2. Consensus Algorithm

The main function of the consensus algorithm is to make all nodes have distributed structure and reach an effective consensus. Its core principles are safety, fault tolerance, and activity. We analyze and compare several commonly used consensus algorithms, and introduce some of their characteristics, as shown in Table 1.

Among them, the calculation operation of POW is as follows:

(1) $$\begin{array}{c} \text{Hash}\left(\text{PreHash,}M\text{root,}T\text{stamp,}N\text{once}\right)<\text{Target}. \end{array}$$

Implementation algorithm of POS:

(2) $$\begin{array}{c} \text{Hash}\left(\text{block\_header}\right)\leq \text{Target}*\text{coinage}. \end{array}$$

PBFT is a practical Byzantine fault-tolerant algorithm. In this paper, we propose to use LS-PBFT without removing centralization, so as to combine with edge computing to make the model more efficient.

(3) $$\begin{array}{c} p=v\mathrm{mod}\left|R\right|, \end{array}$$

(4) $$\begin{array}{c} \langle \langle P,v,n,d\rangle ,m\rangle ,P\in \left\{\text{PRE\_PREPARE, PREPARE, COMMIT}\right\}, \end{array}$$

(5) $$\begin{array}{c} m=m*II\left\{P=\text{PRE PREPARE}\right\}. \end{array}$$

Among them, the view number is represented by *v*, which can express the status of leader nodes; |R| represents the total number of replica nodes and master nodes in the collection. Formulas (3) and (4) are the messaging normal form of nodes. P refers to the message type; *v* represents the message view; *n* represents the message sequence number; *d* represents the message index; *m* is the concrete expression of the content of the message itself. In the process of message delivery, *m* is passed to other nodes only in the first stage, and *m* is not passed again in other stages.

### 2.3. Group Intelligence Perception

“Crowdsensing” [24]: it originated from the use of sensing devices in the Internet of Things. At first, the service purpose of group intelligence perception was only for individuals. However, with the increment of information and the popularity of the Internet, because of the high cost of sensors, people prefer to collect sensing data from mobile phones, computers, and other terminal devices and upload them to servers in the cloud. It can be provided to the party in need. Generally speaking, for different tasks, the perceived time can change, and the perceived data quality is also different. Swarm intelligence sensing technology can process data on a large scale and analyze the quality of sensing data with low cost and quick access.

#### 2.3.1. Incentive Mechanism

In this paper, a user-centered strategy is introduced to obtain perceptual information. Participants provide data and optimization goals that RADP needs to consider the following:

(6) $$\begin{array}{c} \text{RADP}:\max\frac{1}{b_{i}}. \end{array}$$

MSensing method [25]: considering the selection of participants, the RADP strategy is improved. Its optimization goal is as follows:

(7) $$\begin{array}{c} \text{MSensing:max }v\left(w\right)\sum_{i\in w}b_{i}. \end{array}$$

MAA utility function [26]is as follows:

(8) $$\begin{array}{c} S\left(x\right)=\sum_{m=1}^{M}\omega_{m}S\left(x_{m}\right),\\ \sum_{m=1}^{M}\omega_{m}=1. \end{array}$$

Then, the optimization objectives of MAA are as follows:

(9) $$\begin{array}{c} \text{MAA}:\max S\left(x_{i}\right),\;i\in \mu . \end{array}$$

If the policy is platform-centric, the optimization function is as follows:

(10) $$\begin{array}{c} \text{GBMC}:\max\frac{W_{i}{}^{\prime }}{b_{i}}, \end{array}$$

(11) $$\begin{array}{c} \text{ISAM}:\max\frac{d_{i}}{b_{i}=\min E{\left(y_{i}^{\prime }-y_{i}\right)}^{2}/b_{i}}, \end{array}$$

where *b*_*i*_ is the user's bidding price; *w* is the chosen winner. And *S*(*x*_*m*_) refers to the utility function of attributes; Generation *w*_*m*_ refers to attribute weight; *M* can represent the number of attributes; *W*_*i*_′ of formula (9) is interpreted as the range that the user can perceive; *y*_*i*_′ and *y*_*i*_ can be interpreted as estimated mean and accurate mean, respectively.

#### 2.3.2. Trust Management

Updatable ways to manage reputation values:

(12) $$\begin{array}{c} r_{i}=\beta r_{i}\left(t\right)+\alpha r_{i}{}^{-},\\ \alpha +\beta =1,\\ r_{i}\left(t\right)\frac{\left(p\left(t\right)+\varepsilon \right)}{\left(p\left(t\right)+n\left(t\right)+\varepsilon \right)}. \end{array}$$

Redefine according to credible value:

(13) $$\begin{array}{c} \text{TSCM}:\max\sum_{i\in w}v_{i}^{r}-\frac{b_{i}}{r_{i}}. \end{array}$$

## 3. Research on Perceptual Integrity of Group Intelligence Based on AWTC-TT

### 3.1. Evaluate Perceived Quality

Data quality includes accuracy, completeness, consistency, and other attributes. Exploring the integrity of perceived data is an important aspect to ensure the quality of information data. When the data in the database is input, it may lead to input errors or invalid data due to various reasons. Therefore, integrity is put forward to ensure that the data of data digital media conforms to the rules. Generally speaking, data integrity mainly refers to accuracy and reliability, and this experiment will be based on these two points. In addition, the integrity of data can be divided into three categories: domain, entity, and reference.

Before formally exploring completeness, it is necessary to establish standards to evaluate the quality of perceived data. The higher the quality of the data collected, the higher the return given; low returns should be given to low-quality data that fail to meet the standards. Data classification and sorting algorithms are used for evaluation. It is worth noting that every evaluation standard should be based on energy data. Use the following data set:

(14) $$\begin{array}{c} D=\left\{D_{1},D_{2},...,D_{m}\right\}, \end{array}$$

(15) $$\begin{array}{c} C=\left\{C_{1},C_{2},...,C_{k}\right\}, \end{array}$$

(16) $$\begin{array}{c} Z=\left\{\left(Z_{1}^{\min},Z_{1}^{\max}\right),\left(Z_{2}^{\min},Z_{2}^{\max}\right),...,\left(Z_{k}^{\min},Z_{k}^{\max}\right)\right\}. \end{array}$$

Among them, formula (14) describes the data collected about the task. Formula (15) represents the criteria used for data quality assessment. Formula (16) represents each quantifiable effective range, which is related to the evaluation value of the evaluation standard. Get the evaluation value:

(17) $$\begin{array}{c} s_{i,j}=f_{j}\left(D_{i}\right). \end{array}$$

The evaluation value matrix is obtained:

(18) $$\begin{array}{c} S=\left[\begin{array}{ccc} s_{1,1} & \ldots & s_{1,k}\\ \vdots & \ddots & \vdots \\ s_{m,1} & \cdots & s_{m,k} \end{array}\right]. \end{array}$$

(1) Classify
Before judging whether the evaluation requirements are met, calculate the deviation degree. Negative correlation:
  (19) $$\begin{array}{c} e_{i,j}=\left\{\begin{array}{cc} \frac{s_{i,j}-z_{j}^{\min}}{z_{j}^{\max}-z_{j}^{\min}}, & z_{j}^{\min}<s_{i,j}<z_{j}^{\max}\\ 1+\omega , & \text{other}. \end{array}\right) \end{array}$$
Positive correlation:
  (20) $$\begin{array}{c} e_{i,j}=\left\{\begin{array}{cc} \frac{z_{j}^{\max}-s_{i,j}}{z_{j}^{\max}-z_{j}^{\min}}, & z_{j}^{\min}<s_{i,j}<z_{j}^{\max}\\ 1+\omega , & \text{other}. \end{array}\right) \end{array}$$
Determine whether the maximum value meets the requirements:
  (21) $$\begin{array}{c} e_{i}^{\max}=\max\left\{e_{i}\right\}=\max\left\{e_{i,1},e_{i,2},...,e_{i,k}\right\}. \end{array}$$
(2) Quality ranking
Single deviation vector:
  (22) $$\begin{array}{c} e_{i}=\left(\theta_{1}e_{i,1},\theta_{2}e_{i,2},...,\theta_{k}e_{i,k}\right),\\ \overset{k}{\underset{j=1}{\sum }}\theta_{j}=1. \end{array}$$
Bias vector of population:
  (23) $$\begin{array}{c} r_{i}={\left(\sum_{j=1}^{k}{\left(\theta_{j}e_{i,j}\right)}^{2}\right)}^{1/2}. \end{array}$$

Sort the data of HD and LD collections according to the result size. The smaller the value, the higher the quality; the larger the value, the worse the quality. This also shows the integrity of the data.

### 3.2. Fusion and Optimization Design

**Figure 1:**
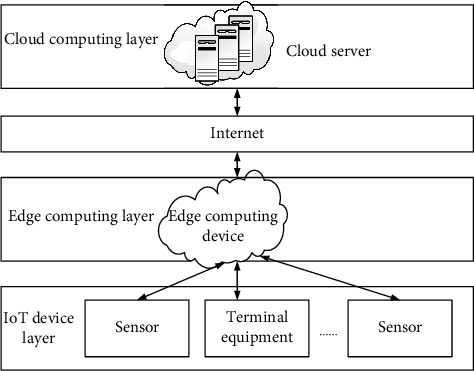
Data sharing architecture.

In order to meet the requirements of digital media diversification and integrate data resources. In order to find out the excellent model and analyze the data integrity, we tried the fusion design of various methods. First of all, we consider that IoT sensors are closely related to terminal devices such as digital media, coupled with the network support of 5G technology. Secondly, in order to improve the transaction speed of each group intelligence-aware service, we choose to join edge computing. This technology can help reduce the pressure on the cloud center, support mobility and meet the high requirements of digital media devices for network delay, and can better sense the location. Its characteristics are in line with the research direction of this subject. In addition, blockchain can be effectively combined with edge computing, and the published perception tasks can be accounted for by blockchain, while edge nodes can cooperate with other nodes to run together. The data-sharing infrastructure is initially established, and a formal hybrid model will be designed based on this architecture, as shown in Figure 1:

### 3.3. Model Building

**Figure 2:**
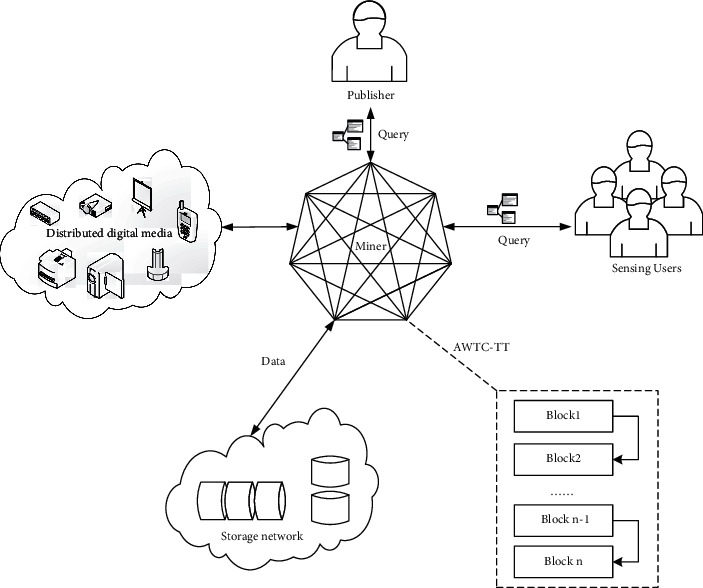
Group intelligence perception hybrid model based on blockchain.

Aiming at the distributed digital media, we publish tasks through the group intelligence sensing network to obtain high-quality sensing data of various terminal devices. Record the transaction information of each perceived service through the blockchain of edge computing. The hybrid model we built is mainly composed of three parts. Blockchain, group intelligence perception, and edge computing are integrated, and the division of labor cooperates with each other. Various terminal devices of digital media upload data; task publishers and perception participants of group intelligence perception query records through blockchain; the blockchain of edge computing serves as a storage network for data transmission, as shown in Figure 2:

## 4. Experimental Analysis

### 4.1. Experimental Setup

Before the simulation experiment, we need to prepare the test environment. Limited by space, we show the most important software and hardware of blockchain here, as shown in Table 2and Blockchain transaction information table is shown in Table 3.

In the fourth round of the AFC Champions League group match, both sides started. Adam3 appeared in China mobile.

Before the simulation experiment begins, it is necessary to preprocess all the collected data. Otherwise, the data are not defined and quantified and cannot be used directly. In the experiment, the task publishers of group intelligence perception released a total of 200 task indicators to perceive the data of digital media terminals.

### 4.2. Comparison of Model Algorithms

**Figure 3:**
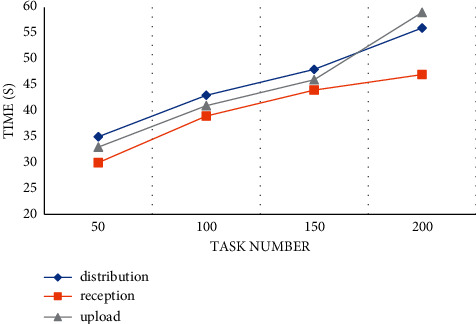
Model transaction time.

First, in this section, we deploy the model architecture and the corresponding test network and conduct data transactions for 200 tasks. First, we confirm the overall data transaction time of the hybrid model. According to the curve showing an upward trend, we can find that the time spent processing transaction tasks increases with the increase in the number of published tasks. This shows that the average time for the algorithm to run and generate accounting blocks is also increasing. Under the limit of 200 tasks, the average processing time of the model is maintained within 1 minute, and the experimental results are acceptable to users, as shown in Figure 3.

**Figure 4:**
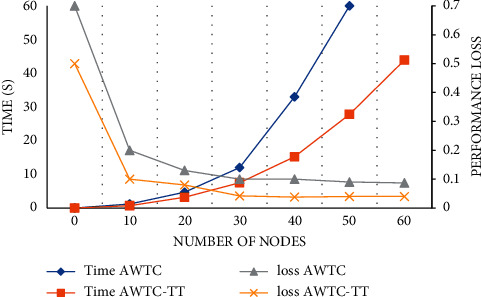
Algorithm performance.

We test the performance of the AWTC-TT algorithm used in the model. Compare the data perception time of the model and the performance loss of the algorithm before and after the application of the optimization algorithm. Nodes package data records into blocks, reach consensus at different edges and connect to the blockchain. The running time of the algorithm is mainly affected by the number of edge nodes. On the whole, the running time of AWTC and AWTC-TT increases with the increase of the number of nodes. However, it can be clearly found that the time of the AWTC-TT algorithm is obviously lower than that of an unoptimized algorithm. The time of the AWTC algorithm in 50 nodes is as high as 1 minute, while the optimization algorithm is only 27.8 s. Until the number of nodes increases to 60, the time spent by the optimization algorithm still does not exceed 50 s, and the highest time can reach 43.9 s. In the comparison of algorithm performance loss, the performance of AWTC-TT is still excellent: in initialization, the performance loss is 20% less than that of AWTC; In the process of formal calculation, the loss rate of AWTC does not increase with the increase of nodes, and basically maintains at about 4%, while the loss rate of AWTC is as high as 10%, as shown in Figure 4.

### 4.3. Perception Result Evaluation

#### 4.3.1. Perceived Quality Assessment

The quality of data perception determines the perception integrity to a great extent. Select the data set of sensing device temperature for the experiment, and let the model randomly generate 100 request tasks. Performance gain, time error, position, and data similarity are analyzed. Here, the performance gain formula is as follows:

(24) $$\begin{array}{c} \text{Gain}=\frac{\sum_{i=0}^{\left[\alpha N\right]}\left(M_{i}^{D}-M_{i}^{T}\right)}{\left[\alpha N\right]}. \end{array}$$

The smaller the error between task time and perceived data measurement time, the higher the reliability. Time error:

(25) $$\begin{array}{c} s_{\text{Time}}=\left|t_{\text{task}}-t_{\text{data}}\right|. \end{array}$$

The difference between the specified node position and the perceived data position: the closer they are, the higher the confidence. Position error:

(26) $$\begin{array}{c} s_{\text{Location}}={\left({\left(H_{\text{task}}-H_{\text{data}}\right)}^{2}+{\left(V_{\text{task}}-V_{\text{data}}\right)}^{2}\right)}^{1/2}. \end{array}$$

The degree of similarity between data determines the reliability of data:

(27) $$\begin{array}{c} s_{\text{Similarity}}=\frac{\sum_{j=1}^{m}\varphi_{i,j}}{m-1},\\ \varphi_{i,j}=\left\{\begin{array}{cc} 1-\frac{\left|\text{val}\left(D_{i}\right)-\text{val}\left(D_{j}\right)\right|}{{\text{val}}_{\max}-{\text{val}}_{\min}}, & i\neq j\\ 0, & i=j. \end{array}\right). \end{array}$$

**Figure 5:**
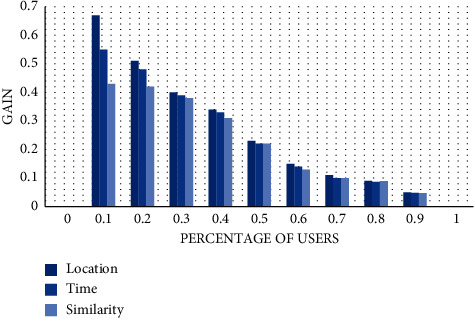
Gain effect of data quality.

We measure the gain value of location, time, and similarity. Here, 0 to 1 of the abscissa represents the percentage index of perceived data. According to the trend of the column chart, we can find that the gain value of 40% of previous data is generally greater than 0.3. However, the gain value of the last 50% to 100% of the perceived data is between 0 and 0.3, which is of low quality. This is because the higher the data quality, the closer the value is to the real result, and the higher is its ranking. In addition, from the chart, we can also find that location's data results have the highest performance advantage, so it is the highest column chart among the three indicators. The second is the time index, which can reach a gain value of 0.55 at the highest; among the three, the weakest advantage is the similarity index, and the highest Gain value is 0.43, as shown in Figure 5:

#### 4.3.2. Data of Abnormal Interference

**Figure 6:**
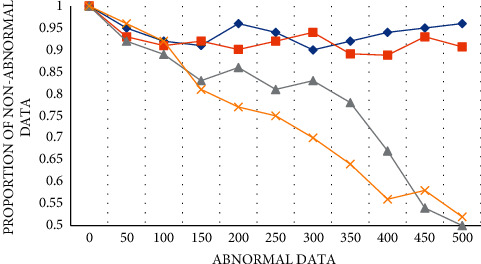
Data interference.

If there is abnormal interference from problematic data in the data, it will also affect the integrity of the perceived data. After quality evaluation, we can successfully judge the high-quality class in the data. We then test the ability of this model method to distinguish abnormal data and test the proportion of non-abnormal data in high-quality data. We set four different digital media application scenarios, *A*, *B*, *C,* and *D*, collected four perceptual data sets, and set the total evaluation value to 1. According to the increase of abnormal data in the four groups in the figure, the curve fluctuation of Group *A* and Group *B* is small, and the proportion of high-quality data is basically maintained at over 90%, which has a good evaluation effect on data integrity. However, the non-abnormal data of Group *C* and Group *D* decreased with the increase of abnormal data, and the curve decreased very quickly, always reducing to about 50%. Generally speaking, the method in this paper has a good effect on the perceptual integrity analysis of digital media. However, there are still some scenes with poor analysis results, as shown in Figure 6:

#### 4.3.3. Perception Result Analysis

**Figure 7:**
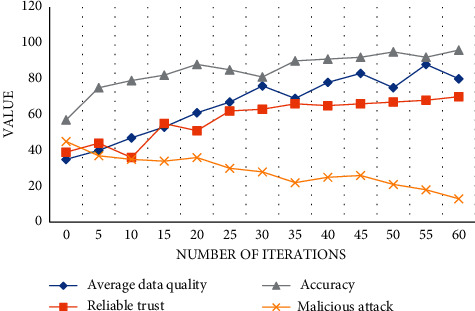
Change trend of perception results.

This experiment is mainly to test the effective integrity of the data in the perception process with the change of iteration times. Let 2000 pieces of data information go through 60 iterations. According to the changes of submitted data, we formulate the changing trend of data integrity. We can find that with the change of iteration times, the average data quality, accuracy, and reliability curve are gradually improved after perceptual processing. Among them, after 60 iterations, the accuracy can be as high as 96%; the reliability and trust of data is about 70%; the average quality of 2000 pieces of data is about 80%. Although the initial fluctuation is large, the data follow-up is relatively stable. However, the curve of a malicious attack on data gradually decreases from 45% to about 13%. To sum up, the data integrity analysis results perceived by the model are very good, as shown in Figure 7:

#### 4.3.4. Perceived Income Distribution

**Figure 8:**
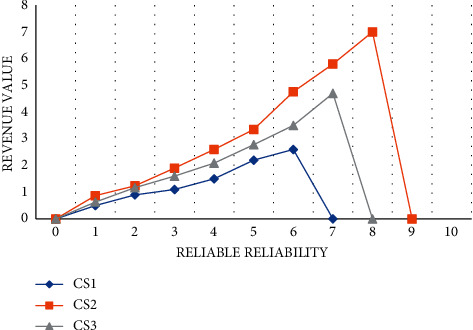
Perceived revenue changes.

We set a reliability value of 0 to 10 and compare the revenue values of CS1, CS2, and CS3. The higher the perceptual quality, the higher the benefit of perceptual distribution. We can find that the maximum return value of CS1 is 2.6; the biggest beneficiary of CS3 is 4.7; the maximum profit value of CS2 is 5.8, and the distribution effect for perceived quality is the best, as shown in Figure 8:

## 5. Conclusion

In recent years, thanks to the rapid development of the Internet of Things and 5G technology, Internet of Things terminal devices are all over people's lives. With the application of digital media, all kinds of data increase rapidly, which brings many challenges to human technology. Based on distributed group intelligence perception, aiming at exploring the integrity performance in the process of data sharing, a model framework with a reasonable structure is constructed, and blockchain technology is applied to practical applications. The idea of this paper focuses on the integrity of perceptual data. Through the experimental results, the feasibility of the proposed method and the feasibility of the AWTC-TT algorithm are verified. According to the characteristics of nodes, this paper solves the problem of deploying blockchain at edge nodes. Increase the number of network nodes, improve the quality of data, and also improve the transaction speed of data sharing. It ensures the fault tolerance of the model, effectively analyzes the performance of simulated data, and improves the calculation efficiency. This article has the above results, but the subject of research is not perfect, and there are still some defects and missing places. In the future research and development direction, there are still many jobs that can be optimized and refined. For example, optimize the parameters and set the model framework more reasonably; try to optimize more algorithms in blockchain technology and explore more technologies for integration; there is no unified experimental standard, so data authentication should be improved; a visual front-end interface should be developed to increase visual effects, so as to build a formal system platform according to the model framework; further optimize and improve data processing performance, reduce the loss of algorithm performance, improve computational efficiency, and meet the application in real application scenarios; data confidentiality should also be carried out for various messages in digital media to ensure security and credibility.

## Figures and Tables

**Table 1:** Compare the performance of the consensus algorithm.

Consensus algorithm name	Characteristics of algorithm	Efficiency	Aggressive resistance	Applicable scenarios
Solo [20]	Single-node consensus model; there is no high availability and scalability	High	Low	Alliance chain or private chain
Kafka [21]	High throughput, low latency, no intentional evil node	High	Low	Alliance chain or private chain
PBFT [22]	Consistency, security, and the number of malicious nodes do not exceed 1/3	High	High	Alliance chain [23]
POW	The hash function is the core; decide who gets out of the block by the calculation difficulty value	Low	High	Public chain
POS	Provide incentive mechanism; it is an improvement of POW	Low	High	Public chain

**Table 2:** Experimental environment.

Environmental configuration of blockchain	Configuration name	Asked anonymously
Hardware environment	CPU	Intel Xeon E5-2682 v4
	RAM	2.00 GB
	SATA	40 GB
	Bandwidth	1.00 MB
	Alibaba Cloud Server (ECS)	3

Software environment	Ubuntu	Easy to live in China
	JAVA	Qiaoxing globe
	JPBC	2.0
	Eosio	Qiaoxing globe
	Eosio.cdt	Qiaoxing globe
	Eosio.tracts	Qiaoxing globe

**Table 3:** Blockchain transaction information table.

Name	Type	Field description	Nonempty	Primary key	Self-increasing	Unique
ID	INT	ID number	Yes	Yes	Yes	Yes
Vendor	TEXT	User name	Yes	No	No	No
Passwd	TEXT	Password	Yes	No	No	No
Type	TEXT	Data type	Yes	No	No	No
Amount	TEXT	Amount of data	Yes	No	No	No
Cost	REAL	Cost price	Yes	No	No	No
Area	TEXT	Acquisition area	Yes	No	No	No
Count	REAL	Balance	Yes	No	No	No

## Data Availability

The experimental data used to support the findings of this study are available from the corresponding author upon request.
